# Adverse events related to accessory devices used during ureteroscopy: Findings from a 10‐year analysis of the Manufacturer and User Facility Device Experience (MAUDE) database

**DOI:** 10.1002/bco2.274

**Published:** 2023-08-09

**Authors:** Patrick Juliebø‐Jones, Bhaskar K. Somani, Ioannis Mykoniatis, B. M. Zeeshan Hameed, Lazaros Tzelves, Mathias S. Æsøy, Peder Gjengstø, Christian Arvei Moen, Christian Beisland, Øyvind Ulvik

**Affiliations:** ^1^ Department of Urology Haukeland University Hospital Bergen Norway; ^2^ Department of Clinical Medicine University of Bergen Bergen Norway; ^3^ EAU YAU Urolithiasis Group Arnhem Netherlands; ^4^ Department of Urology University Hospital Southampton Southampton UK; ^5^ School of Medicine, Department of Urology, Faculty of Health Sciences Aristotle University of Thessaloniki Thessaloniki Greece; ^6^ Department of Urology Father Muller Medical College Mangalore Karnataka India; ^7^ Second Department of Urology National and Kapodistrian University of Athens, Sismanogleio General Hospital Athens Greece

**Keywords:** basket, ureteral access sheath, ureteroscopy, urolithiasis

## Abstract

**Objectives:**

The objective of this study was to evaluate adverse events and device events related to accessories used during ureteroscopy (URS).

**Materials and methods:**

Analysis was performed of the records available in the Manufacturer and User Facility Device Experience database in the United States. Information was collected on characteristics of problem, timing, manufacturer verdict, successful completion of planned surgery, prolonged anaesthesia and injury to patient or staff.

**Results:**

Five‐hundred seventy‐one events related to URS accessories were recorded. These were associated with the following devices: baskets (*n* = 347), access sheath (*n* = 86), guidewires (*n* = 78), balloon dilators (*n* = 27), ARDs (*n* = 17) and ureteral catheters (n = 16). Of the events, 12.7% resulted in patient injuries. Forty‐eight per cent of the events resulted in prolonged anaesthesia, but the planned surgery was successfully completed in 78.4% of all cases. Collectively, the manufacturers accepted responsibility due to actual device failure in only 0.5% of cases. Common problems for baskets were failure to deploy (39.5%) and complete detachment of basket head (34.6%) and partial breakage of the basket head (12.4%). Of the basket group, 4.3% required open or percutaneous surgery to remove stuck basket. Full break of the body of the access sheath occurred in 41.9% and complete ureteral avulsion in 3.5%. For balloon dilators, there was a burst in 37% of cases. Broken guidewires were associated with 11.5% requiring repeat intervention for retrieval and 6.4% required JJ stent due to perforation to the collecting system. No injuries to operating staff were recorded with accessory usage.

**Conclusion:**

Accessories used during URS are fragile. Potential for serious injury does exist as a direct result of their use. Surgeons should familiarise themselves with these events and how they can be prevented.

## 1 INTRODUCTION

The plethora of accessories, which are available to the surgeon when performing ureteroscopy (URS), is one of the key reasons for its success as a minimally invasive treatment choice for urolithiasis.^1^, ^2^, ^3^ Amongst other qualities, the ideal accessory instrument should be durable, atraumatic and ergonomic for the user. The pursuit to deliver improved patient outcomes has led to innovations and technological advancements for all such devices.^4^ However, none of the instruments can be considered perfect, and limitations still persist. To this end, intra‐operative complications during URS do continue to occur due to events related to their use.^5^ New modifications to established devices are generally accompanied by studies evaluating their efficacy, safety and durability.^6^ While these offer valuable insight, the setting is nearly always ex vivo rather than in vivo.^7^, ^8^ The clinical burden of device‐ and accessory‐related adverse sequelae in real world practice remains limited. The Manufacturer and User Facility Device Experience (MAUDE) database in the United States is a prospectively maintained library of anonymously reported failures related to surgical devices.^9^ This inventory of adverse events can serve to provide valuable learning lessons across a wide range of surgical disciplines.^10^ While there are a number of studies examining findings relevant to urological surgery, to our knowledge, there are none dedicated to this category of instruments and accessories used so widely in endourology.

Our aim was to perform an evaluation of URS‐related accessories and gain an overview of the adverse events that have been reported over a 10‐year period.

## 2 MATERIALS AND METHODS

A search was performed for accessories related to URS that were contained in the MAUDE database between 2012 and 2021. Of note, this public database is strictly limited to cases from the United States only. It is managed by the US Food and Drug Administration and serves the purpose of monitoring the safety of approved devices.^9^ It was established in its earliest form in 1993. Data can be added by health professionals and medical companies alike. Reporting is both voluntary and anonymous.

The following accessories were included: stone baskets, stone anti‐retropulsion devices (ARDs), ureteral access sheaths (UASs), balloon dilators, ureteric catheters and guidewires. A report was excluded if it was judged to be of insufficient quality or lacked information. Duplicate reports were also excluded. Information was collected on characteristics of problem, timing, manufacturer verdict, successful completion of planned surgery, prolonged anaesthesia and injury to patient or staff.

All information was both anonymous and publicly available, and therefore, ethical approval was deemed not to be required.

## 3 RESULTS

Over the 10‐year study period, a total of 571 events related to URS accessories were recorded. These were associated with the following devices: baskets (*n* = 347), UAS (*n* = 86), guidewires (*n* = 78), balloon dilators (*n* = 27), ARDs (*n* = 17) and ureteral catheters (*n* = 16) (Tables 1 and 2). These were produced by 11 different manufacturers. While no injuries to surgeon or operating staff were recorded, 12.7% of the events resulted in patient injuries. Forty‐eight per cent of the events resulted in prolonged anaesthesia, but the planned surgery was successfully completed in 78.4% of all cases. Collectively, the manufacturers accepted responsibility due to actual device failure in only 0.5% of cases.

**TABLE 1:** Summary of results for baskets, anti‐retropulsion devices and guidewires.

Accessory	Total No.	Problem	Identified timing				Anaesthesia prolonged	Procedure successfully completed
			Pre URS	During URS	Post URS	Later surgery		
Basket	347	Not deploying: 137 (39.5%) Broke off completely: 120 (34.6%) Partial break: 43 (12.4%) Handle snapped: 14 (4%) Basket stuck: 11 (3.2%) Not closing: 6 (1.7%) Basket found at later case: 2 (0.6%) Missing part: 2 (0.6%) Contaminated: 1 (0.3%) Cable snapped: 1 (0.3%)	87 (25.1%)	256 (73.7%)	2 (0.6%)	2 (0.6%)	148 (42.7%)	247 (82.7%)
ARD	17	Detached: 11 (64.7%) Not deploying: 2 (11.8%) Flaking of coating: 2 (11.8%) Damaged: 1 (5.9%) Stuck: 1 (5.9%)	2 (11.8%)	10 (58.8%)	2 (11.8%)	3 (17.6%)	10 (58.8%)	12 (70.6%)
Guidewire	78	Flaking of coating: 22 (28.2%) Tip broke off: 19 (24.4%) Broke in half: 17 (21.8%) Coating stripped off: 11 (14.1%) Stuck in patient: 4 (5.1%) Perforated collecting system: 4 (5.1%) Found part at later case: 1 (1.3%)	2 (2.6%)	72 (92.3%)	3 (3.8%)	1 (1.3%)	23 (29.5%)	68 (87.2%)

Abbreviation: ARD, anti‐retropulsion device.

**TABLE 2:** Summary of results for ureteral access sheaths, balloon dilators and ureteral catheters.

Accessory	Total No.	Problem	Identified timing				Anaesthesia prolonged	Procedure successfully completed
			Pre URS	During URS	Post URS	Later surgery		
Ureteral access sheath	86	Break to body: 36 (41.9%) Ureteral injury: 14 (16.3%) Sheath split: 10 (11.6%) Flaking of coating: 9 (10.5%) Inner sheath damaged: 7 (8.1%) Inner sheath not deploying: 5 (5.8%) Tip broke off: 4 (4.7%) Scope stuck in sheath: 1 (1.2%)	6 (7%)	78 (90.7%)	1 (1.2%)	2 (2.3%)	56 (65.1%)	63 (73.3%)
Balloon dilator	27	Burst: 10 (37%) Not inflating: 5 (18.5%) Leaking balloon: 4 (14.8%) Not deflating: 2 (7.4%) Broke in half: 1 (3.7%) Faulty: 3 (11.1%)	4 (14.8%)	23 (85.2%)	0	0	22 (81.5%)	25 (92.6%)
Ureteral catheter	16	Tip broke off: 12 (75%) Flaking coating: 1 (6.3%) Kinked: 1 (6.3%) Perforated ureter: 1 (6.3%) Contaminated: 1 (6.3%)	2 (12.5%)	13 (81.3%)	1 (6.3%)	0	4 (25%)	14 (18.8%)

### 3.1 Stone baskets

The commonest problems were failure to deploy (39.5%), complete detachment of basket head (34.6%) and partial breakage of the basket head (12.4%). While the majority of cases were still successfully completed (82.7%), over one third (42.7%) resulted in prolonged anaesthesia to rectify the problem. There were two cases of retained baskets being identified in the kidney at the time of a later URS for new stone episodes. While only 6.9% of the events caused patient injury, these were often serious and included, retained basket requiring open surgery or percutaneous access (4.3%), ureteric perforation on exiting the kidney (1.4%) and complete ureteral avulsion (<1%) (Table 3). One case was reported of basket and scope becoming fully entrapped. Open surgery and removal were performed on the following day to resolve this.

**TABLE 3:** Summary of patient injuries

Device	Number of patient injuries	Injury type and management
Basket	24 (6.9%)	Retained part: 15 (4.3%)—surgery (open or PCNL/antegrade URS) Ureteral perforation: 5 (1.4%)—JJ stent Avulsion: 2, (0.6%)—open surgery Retained scope & basket: 1 (0.3%)—open surgery Bleeding: 1, (0.3%)—early termination of case
ARD	4 (23.5%)	Retained part: 4 (23.5%)—surgery (PCNL)
Guidewire	15 (19.2%)	Retained fragment: 9 (11.5%)—repeat surgery (URS/IR) Collecting system perforation: 5 (6.4%)—JJ stent Bleeding: 1 (1.3%)—early termination of case
Ureteral Access Sheath	21 (24.4%)	Ureteric perforation: 10 (11.6%)—JJ stent Retained parts 4 (4.7%)—repeat surgery (URS) Bleeding: 3 (3.5%)—early termination of case Avulsion: 3 (3.5%)—emergency open surgery Wrong side surgery: 1 (1.2%)—repeat surgery (URS)
Balloon dilator	4 (14.7%)	Ureteral perforation: 4 (14.8%)—JJ stent
Ureteral catheter	2 (12.5%)	Ureteric perforation: 1 (6.3%)—JJ stent Retained part: 1 (6.3%)—surgery (URS)

Abbreviations: IR, interventional radiology; PCNL, percutaneous nephrolithotomy; URS, ureteroscopy.

### 3.2 Ureteral access sheath

A full break of the body of the UAS was the most frequently reported problem (41.9%). In 11.6%, the sheath was torn along its length, while detachment isolated to the tip was reported in 4.7%. The inner sheath was either damaged or not deploying in 8.1% and 5.8% of cases, respectively. These events resulted in prolonged anaesthesia in over half of the surgeries (65.1%), but most (73.3%) could still be completed. Detachment of a part of UAS resulted in repeat URS for retrieval in 4.7%. The most serious injury was complete ureteral avulsion necessitating open surgery for repair (3.5%). This included a case of wrong side surgery requiring repeat URS to be performed but on the correct side.

### 3.3 Guidewires

The main problem was breakage of the guidewire. This was either limited to only the tip (24.4%) or involved the main body (21.8%). Overall, 11.5% required repeat intervention (via URS or interventional radiology) to retrieve the retained part. A large proportion (42.3%) involving guidewires were related to their coating. In 28.2%, this was flaking off gradually, and in 14.1%, it was completely stripped off when passing through the scope. The latter carried more potential for harm due to exposure of the sharp inner metal core, and one case had to be terminated due to bleeding associated with this event. Events nearly always occurred during the URS procedure (92.3%). Overall, 6.4% required JJ stent due to perforation to the collecting system related to the faulty guidewire.

### 3.4 Balloon dilators

Over one third of events (37%) were related to burst balloon, while in 18.5% and 7.4%, it would not inflate or deflate, respectively. Such problems resulted in a high proportion of cases requiring a prolonged anaesthetic time (81.5%). Only in one case did the device break in half while in use. The only type of patient injury reported was ureteral perforation (14.8%) requiring insertion of JJ stent.

### 3.5 Anti‐retropulsion devices

Detachment of the ARD occurred in 64.7% of the events in this category. When identified intra‐operatively, these were resolved successfully albeit incurring additional anaesthesia time. In one case, the whole device became completely stuck requiring percutaneous approach with antegrade removal. This method was also required in three other cases where retained parts from original surgery had become lodged, and this was found at the time of subsequent surgery.

### 3.6 Ureteral catheters

In comparison to other accessory types, there were relatively few issues for this particular device. Three quarters of issues involved the tip breaking off. Only in one case could this fragment not be retrieved at the time of surgery. Of the problems identified before URS was commenced, one included a contaminated device involving human hair found in the packaging. Out of all these events involving ureteral catheters, one caused ureteral perforation and the patient received a JJ stent accordingly.

## 4 DISCUSSION

This study highlights the wide range of potential problems that can occur with accessories used commonly in daily URS practice. While many of these resulted in prolonged anaesthesia, most cases were still successfully completed. However, the potential exists for serious injury needing percutaneous removal and endourological nightmare situations such as complete ureteral avulsion as in the case of entrapped accessories and scopes.

An awareness of the potential adverse events associated with accessories that can occur during URS is highly relevant given the increasing volume of surgeries being performed. Haas et al.^11^ reported that 21 184 URS were performed annually in the United States while in 2019, it had increased to over 60 000.

The potential risks associated with certain devices such as UAS and balloon dilators are widely reported.^12^, ^13^, ^14^ Indeed, these form part of the common arguments against their use. However, our study also spotlights those injuries, which can be associated with instruments that are typically presumed to be relatively innocuous such as the guidewire. Our findings highlight the need for surgeons to have the knowledge and an understanding of potential device issues that can occur, as well as their management. We found that the manufacturers claimed a device failure in a very low number of cases (<1%). It is acknowledged that, in such instances, it is extremely difficult to discern the true cause at a later date when such fragile instruments have been used in an operation. The top cause suggested by the manufacturers was excess force used by the surgeon. Our study serves as a reminder of the mantra that the use of force should be avoided in endourology. The focus of simulation skills in endourology tends to be on use of the scope.^15^ Use of accessories should also be central to this. On a practical basis, replacement equipment should be stocked and be readily available, given how damage to instruments and accessories can frequently occur.

## 5 LIMITATIONS

Limitations to acknowledge from our study are largely those associated with the MAUDE database. It is reliant on voluntary reporting, and clinicians are not obliged to report device failures. To this end, no estimates of the possible incidence of such events can be reliably calculated, as highlighted in a previous study by Friedman et al.^16^ However, it does seem that the number of events is very low compared to the high number of cases performed in the United States during the study period. The authors would anticipate that if reporting was mandatory, the true number of events would be much higher. The database also lacks information related to parameters such as patient demographics, surgeon experience and hospital setting. More detailed information on prolonged anaesthesia was lacking in the database, and this is a further limitation.

While we have included six categories of accessories, there are other important items in endoscopic surgery such as ureteral stents that have not been covered in this study, and this can also be considered a drawback.

However, the findings from this database reveal a ‘blackhole’ of repeat device related events, which are known but scarcely reported in the literature. Unless such information becomes a routine supplement to the standard parameters measured in studies, this ‘under the radar’ catalogue of events is unlikely to be documented outside of databases such as MAUDE, which is the largest of its kind. In this regard, it carries a unique strength despite the aforementioned weaknesses. This is especially the case, since other databases such as the Rockwell Laser database have ceased to contain such clinical information. Similar databases need to be established elsewhere in the world to capture the true nature of these adverse events, leading to better surgeon awareness and training and safer surgeons of tomorrow. Further studies on the database assessing events related to other equipment used in endoscopic stone surgery such as lasers and ureteral stents would also be valuable.

## 6 CONCLUSIONS

Accessories used during URS are fragile, and a wide range of problems can occur. The potential for serious injury does exist as a direct result of their use. Surgeons should familiarise themselves with these events and strategise to avoid them.

## AUTHOR CONTRIBUTIONS

**Patrick Juliebø‐Jones:** Conception; data collection; analysis; writing of draft and revision. **Bhaskar K. Somani:** Conception; data collection; analysis; writing of manuscript. **Ioannis Mykoniatis:** Data collection; analysis; writing of manuscript. **B. M. Zeeshan Hameed:** Data collection; analysis; writing of manuscript. **Lazaros Tzelves:** Data collection; analysis; writing of manuscript. **Mathias S. Æsøy:** Data collection; analysis; writing of manuscript. **Peder Gjengstø:** Conception; analysis; writing of manuscript. **Christian Arvei Moen:** Data collection; analysis; writing of manuscript. **Christian Beisland:** Data collection; analysis; writing of manuscript; supervision. **Øyvind Ulvik:** Data collection; analysis; writing of manuscript; supervision.

## CONFLICT OF INTEREST STATEMENT

Øyvind Ulvik has acted as a consultant for Olympus. The other authors have conflict of interest to declare.
